# Stochastic gradient descent optimisation for convolutional neural network for medical image segmentation

**DOI:** 10.1515/biol-2022-0665

**Published:** 2023-08-08

**Authors:** Sanam Nagendram, Arunendra Singh, Gade Harish Babu, Rahul Joshi, Sandeep Dwarkanath Pande, S. K. Hasane Ahammad, Dharmesh Dhabliya, Aadarsh Bisht

**Affiliations:** Department of Artificial Intelligence, KKR & KSR Institute of Technology and Sciences, Guntur, India; Department of Information Technology, Pranveer Singh Institute of Technology, Kanpur, 209305, Uttar Pradesh, India; Department of E.C.E, CVR College of Engineering, Hyderabad, India; CSE Department, Symbiosis Institute of Technology, Symbiosis International (Deemed University), Pune, India; MIT, Academy of Engineering, Alandi, Pune, India; Department of E.C.E., Koneru Lakshmaiah Education Foundation, Vaddeswaram, 522302, India; Department of Information Technology, Vishwakarma Institute of Information Technology, Pune, India; University Institute of Engineering, Chandigarh University, Mohali, India

**Keywords:** machine learning, convolutional neural networks, medical chest-X-ray images, SGD

## Abstract

In accordance with the inability of various hair artefacts subjected to dermoscopic medical images, undergoing illumination challenges that include chest-Xray featuring conditions of imaging acquisi-tion situations built with clinical segmentation. The study proposed a novel deep-convolutional neural network (CNN)-integrated methodology for applying medical image segmentation upon chest-Xray and dermoscopic clinical images. The study develops a novel technique of segmenting medical images merged with CNNs with an architectural comparison that incorporates neural networks of U-net and fully convolutional networks (FCN) schemas with loss functions associated with Jaccard distance and Binary-cross entropy under optimised stochastic gradient descent + Nesterov practices. Digital image over clinical approach significantly built the diagnosis and determination of the best treatment for a patient’s condition. Even though medical digital images are subjected to varied components clarified with the effect of noise, quality, disturbance, and precision depending on the enhanced version of images segmented with the optimised process. Ultimately, the threshold technique has been employed for the output reached under the pre- and post-processing stages to contrast the image technically being developed. The data source applied is well-known in PH^2^ Database for Melanoma lesion segmentation and chest X-ray images since it has variations in hair artefacts and illumination. Experiment outcomes outperform other U-net and FCN architectures of CNNs. The predictions produced from the model on test images were post-processed using the threshold technique to remove the blurry boundaries around the predicted lesions. Experimental results proved that the present model has better efficiency than the existing one, such as U-net and FCN, based on the image segmented in terms of sensitivity = 0.9913, accuracy = 0.9883, and dice coefficient = 0.0246.

## 1 Introduction

Medical images have advanced due to the rapid advancement of digital technology in all areas of life. Most images are medically divided with the digital and formatting approach for the analysis [1]. Based on the challenges faced over the province of images that influence with the process of the optimised method of requesting information being structured to the principal analyst for the magnetic resonance imaging (MRI) scans subjected to the control focused into the process of computerised scam developed [2]. Further, the automatic process’s physical condition establishes a precise optimisation analysis. They additionally diagnose the automated accounting for the information passed to the clinical approach being developed into the registries with the importance of chest-Xray medical images. Several modalities that include X-ray, MRI, CT, microscopy, single photon emission computed tomography, positron emission tomography (PET) endoscopy, optical coherence tomography, and so on utilizing the creation of images clinically applied with the patients organised into body parts with separate existence of featuring image segmentation assist to the boundaries of the simulation with the simulation mounted to the insights developed under gaining the additional strategy to the region related to the diagnosis [3,4]. As per the challenges faced by the images that associate with the process of optimised approach of requesting information being structured to the principal analysis for the MRI scans subjected to the control focused into the process of CT scan developed feature learning, classifier level, and decision making [5]. Numerous approaches for image segmentation featuring enable to assist the boundaries of the simulation that is mounted to the insights developed is presented under gaining the additional strategy to the region related to the diagnosis brain image segmentation iterative deep neural network structure for medical image segmentation [6,7]. Moreover, the method utilizes an encoder–decoder system integrated an iterative design until they can improve the segmentation results in medical images that contain complex shapes recursive optimised into objective function fed to the case of appropriate functioning with the characteristics smoothing to the system provided encoder–decoder structure with 64 layers (excluding the final activation layer). Before initial sampling begins, two dense layers are present in the network’s centre [8]. Convolutional neural network (CNN) segments six textures in MRI brain images, chest muscle in MRI images, and vessels in CT cardiac images. Hence, this method is appropriate for segmenting different organs and imaging conditions [9]. Each encoder sequence has attained numerous convolutional layers under batch normalised integrated to ReLU nonlinearity observed through a sampling technique designed with the non-overlapping case for the max pooling and sub-sampling strategy. Before initial sampling begins, two dense layers are present in the network’s centre for speeding up the neural network learning process by normalizing the values in the hidden layers, like the principle behind the normalisation of features in data or activation values. This method has the potential to outperform two fully convolutional networks (FCN) and U-net architectures. Despite using up-convolutional layers and a few shortcut connections, FCN generates coarse segmentation maps. As a result, more shortcut connections are added [10]. In contrast to FCN, indices from max-pooling are copied rather than encoder features. This proposed method uses less memory than FCN. Also, the outputs produced by this method are smooth, and the borders between the region of interest (ROI) and the surrounding areas cannot be detected precisely by the physicians; therefore, we used a threshold technique as a post-processing technique to sharpen the results. The batch normalisation layer is presented after each convolution layer in the proposed network for 25 batch normalisation layers in the entire network architecture. To avoid overfitting, they used two techniques: transfer learning and data augmentation. These methods are classified into non-automatic, semi-automatic, and automatic. Because there is no human intervention in automated processes, the possibility of error is reduced.

Researchers and practitioners have been working on addressing these challenges and developing advanced optimisation algorithms and techniques that build upon stochastic gradient descent (SGD), such as momentum, adaptive learning rate methods (e.g. AdaGrad, RMSprop, Adam), and second-order optimisation methods (e.g. L-BFGS, conjugate gradient methods). These approaches aim to improve convergence speed, stability, and robustness to different problem settings.

SGD is a popular optimisation method used in CNNs for medical image segmentation. It works by updating the weights in the network based on the partial derivatives of the loss function with respect to each weight. This process is repeated iteratively for multiple epochs to minimise the loss and improve the accuracy of the model. In particular, SGD is a stochastic optimisation method, meaning that it uses a subset of the training data (known as a mini batch) to compute the gradients and update the weights at each iteration. This approach can be more efficient than using the entire training set, especially for large datasets. It is a variation of the gradient descent method, but instead of computing the gradient of the cost function over the entire dataset, it randomly selects a single data point (or a small subset of the data) at each iteration and computes the gradient based on that data point. This makes SGD much faster and more scalable than standard gradient descent, especially for large datasets. Additionally, SGD can help to avoid getting stuck in local minima by introducing randomness into the training process. Additionally, CNNs are a popular type of neural network for image segmentation tasks because they can learn to extract features from images that are relevant for the task at hand. This allows them to identify boundaries between different regions of the image and produce accurate segmentation maps. CNN involves using a deep learning model that learns to automatically identify and classify the different structures within an image. The CNN method is particularly useful for medical image segmentation because it can handle complex and heterogeneous structures, and it can adapt to different imaging modalities and resolutions. Additionally, CNNs can be trained on large amounts of data, which is important for accurate segmentation. To use the CNN method for medical image segmentation, one typically needs to prepare the training data, design the network architecture, and train the model on the data. This can involve many steps and requires expertise in deep learning and medical imaging. Certainly, CNNs and SGD are two popular methods for medical image segmentation. CNNs are a type of deep neural network commonly used for image classification and segmentation tasks. They are known for their ability to automatically extract and learn features from images, which makes them an effective method for segmentation. CNNs work by applying a series of convolutional filters to the input image, gradually learning complex patterns that help identify the ROI. SGD, on the other hand, is an optimisation algorithm commonly used to train machine learning models. It works by iteratively adjusting the weights of the model to minimise the difference between predicted and actual outputs. In the context of medical image segmentation, SGD can be used to train a variety of models, including CNNs. It is worth noting that while SGD offers these benefits, it also has some limitations and challenges, such as the sensitivity to learning rate choice, convergence to suboptimal solutions, and potential difficulties in handling noisy or unbalanced data. However, researchers have developed numerous extensions and variations in SGD to address these challenges, making it a powerful and widely used optimisation algorithm in the machine learning community.

A medical image segmentation technique based on an optimised CNN with adaptive dropout depth calculation is proposed in “Automatic data augmentation for 3D medical image segmentation” in Medical Image Computing and Computer Assisted Intervention. The study also investigates how deep neural networks’ architectures might be learned through differential evolution for analysing medical images [11,12,13].

Overall, both CNN and SGD have their own strengths and weaknesses when it comes to medical image segmentation. CNNs are generally better suited for tasks involving large amounts of data and complex image structures. SGD can be used with a wide range of models and is particularly effective when working with smaller datasets. Ultimately, the choice of method will depend on the specific needs of the project at hand. Medical image segmentation is an active area of research in the field of medical imaging. There are several ongoing research efforts aimed at developing new and improved methods for segmenting medical images. Some of the current research areas in medical image segmentation include: (a) Deep learning-based segmentation methods: Deep learning methods such as CNNs have shown promising results in medical image segmentation. Researchers are exploring different architectures and training regimes to improve the accuracy and robustness of these methods. (b) Multi-modal segmentation: Medical images often consist of multiple modalities, such as CT and MRI scans. Researchers are developing new methods to combine information from multiple modalities to improve segmentation accuracy and reduce errors. (c) Interactive segmentation: Interactive segmentation methods allow clinicians to interact with the segmentation algorithm to refine the results. Researchers are exploring new ways of integrating feedback from clinicians to improve the accuracy and efficiency of the segmentation process. (d) Domain adaptation: Medical images can vary significantly depending on the imaging modality, patient population, and other factors. Researchers are developing methods for adapting segmentation algorithms to new imaging domains to improve generalisation and reduce the need for large amounts of training data. Overall, medical image segmentation remains an active and exciting area of research, with many opportunities for innovation and improvement.

The performance metrics depends on the specific segmentation task, the target structure, and the evaluation criteria defined in the research or application context. CNNs and their variants, such as U-Net, SegNet, and DenseNet, have been widely used for medical image segmentation due to their ability to learn complex patterns and hierarchical representations. Researchers have explored architectures and modifications to improve segmentation accuracy, including skip connections, residual connections, attention mechanisms, and spatial context aggregation modules. Attention mechanisms: Attention mechanisms have gained popularity in medical image segmentation to selectively focus on relevant regions or features. Techniques such as channel attention, spatial attention, and self-attention mechanisms have been applied to improve the localisation and segmentation of target structures. Generative adversarial networks (GANs): GANs have been utilised for medical image segmentation to generate realistic and accurate segmentation masks. Adversarial training frameworks, such as Conditional GANs (cGANs) and pix2pix, have been used to enhance the quality of segmentation outputs. Multi-modality fusion: Integration of information from multiple imaging modalities, such as MRI, CT, PET, or ultrasound, has been explored to improve segmentation performance. Fusion techniques, including early fusion, late fusion, and multi-branch architectures, have been investigated to leverage complementary information from different modalities. Uncertainty estimation: Estimating uncertainty in medical image segmentation is important for assessing the reliability of the segmentation results [14,15,16,17,18]. Bayesian methods, dropout sampling, Monte Carlo sampling, and ensemble-based techniques have been applied to estimate uncertainty and provide confidence measures for segmentation predictions.

Structure of the research article is as follows: Section 2 describes the related works associated with the medical image segmentation. Section 3 presents the proposed methodology. Section 4 deals with the network architecture. Image augmentation process with the network configurations for the training, testing, and validation is explained in Section 5. Section 6 contains experimental data, its results, and design. Section 7 gives the comparative analysis. Section 8 demonstrates the results and discussions followed by conclusion in Section 9.

Convergence times for SGD can be lengthy, particularly when working with huge datasets or intricate models. In contrast to other optimisation techniques, SGD only updates the model parameters based on a portion of the training data, which introduces noise and may cause delayed convergence. SGD’s performance depends greatly on the learning rate that is selected. Instable training or divergence can come from an excessive learning rate, whereas sluggish convergence might be caused by a low learning rate. Finding the ideal learning rate can be difficult and sometimes calls for manual tuning or the use of adaptive learning rate techniques. When working with sparse datasets, where most of the features have zero or extremely few values, SGD may run into problems. In flat or saddle portions in the loss landscape, when the gradient is nearly nil, SGD may have difficulty escaping. These areas can drastically slow down the algorithm’s convergence. To address this issue, researchers have suggested modifying SGD by including momentum or employing second-order optimisation techniques. Particularly when working with complicated models and scant training data, SGD is prone to overfitting. When a model performs well on training data but struggles to generalise to untried data, overfitting takes place. To deal with this issue, regularisation strategies like L1 or L2 regularisation, dropout, or early halting are frequently used.

## 2 Related works

As stated, [9], the physician requires many medical sources for clinical handling to develop précised rates with statistical information and patient analysis. Many computer-based algorithms have been developed in recent years to solve the problem of medical image segmentation. Among these initiatives is an iterative deep neural network structure for medical image segmentation. This method combines an encoder–decoder design with an iterative system until they can improve the segmentation results in medical images containing complex shapes. To avoid overfitting, they used two techniques: transfer learning and data augmentation. The other work proposed a method for segmenting cardiac medical images [19]. First, a YOLO neural network detects ROI, which is fed into a CNN for segmentation. Finally, a fully connected neural network classifies the segmented images, allowing the doctor to detect precisely the type of cardiac disease. Various methods demonstrated that represent for featuring image segmentation to assist the boundaries of the simulation with the simulation mounted to the insights developed under gaining the additional strategy to the region related to the diagnosis of brain image segmentation [3]. These methods are classified into non-automatic, semi-automatic, and automatic. Because there is no human intervention in automated processes, the possibility of error is reduced. Encoder–decoder structure has 64 layers (excluding the final activation layer). Each encoder sequence has attained numerous convolutional layers under batch normalised integrated to ReLU nonlinearity observed through a sampling technique designed with the non-overlapping case for the max pooling and sub-sampling strategy. Before initial sampling begins, two dense layers are present in the network’s centre.

Another approach could train a CNN to segment six textures in MRI brain images, chest muscle in muscle MRI images, and vessels in CT cardiac images. Hence, this method is appropriate for segmenting different organs and imaging conditions. Abdelhafiz et al. proposed a technique for addressing mammographic lesions using U-net neural networks [6]. In this work, batch normalisation and data augmentation improved accuracy. Fu et al. [5] developed a method for segmenting eye vessels using fully connected neural networks. This network generates an eye vessel feature map and feeds it to the CRF neural network, which generates binary images of the eye vessels. Guo et al. [7] presented the research work which examine the medical images at three levels: Challenges with the field into the province of images that are challenging with the process of optimised approach of requesting information being structured to the principal analyst for the MRI scans subjected to the control focused into the process of CT scan developed feature learning, classifier level, and decision making. They created a neural network to detect malignant tumours in various imaging conditions (CT, PET, and MRI). Emre Celebi et al. [8] developed a method for detecting skin lesions using four threshold methods. Zhou et al. [9] normalized the values in the hidden layers, like the principle behind normalizing features in data or activation values for speeding up the neural network learning process. The batch normalisation layer is presented after each convolution layer in the proposed network. Twenty-five batch normalisation layers in the entire network architecture used mean shift estimation to improve automatic skin lesion segmentation. The technique altered with a vital image placed in the appropriate precise to maintain exact orientation with the image including a mask of truth nature throughput. Many computational sequences are required for this method. Xie and Bovik [10], combined a neural network method with a genetic algorithm to segment skin lesions. CNNs have recently emerged as one of the most essential and practical algorithms for image segmentation, particularly for medical image segmentation [19]. Its algorithm has been used for brain tumour segmentation in MRI brain images [20], cardiac image segmentation [21], skin lesion segmentation using non-dermoscopic images [22], and many other medical image segmentation tasks involving different parts of the body, different organ shapes, and various artefacts.

## 3 The proposed method

With estimate of the applied knowledge in application of stochastic medium built with gradient descent optimisation because substitutes to the original division of gradient analysis through estimation strategy. The optimisation approach is utilised with SGD practice. It is an iterative procedure with the recursive optimised into objective function fed to the case of appropriate functioning with the characteristics smoothing to the system provided.

SGD is an optimisation algorithm used to minimise the loss function in machine learning. It works by iteratively updating the model parameters in small batches of data, instead of using the entire dataset at once. The algorithm works as follows:

1. Initialise the model parameters with random values.
2. Choose a random subset of the training data (called a mini batch) to feed into the model.
3. Compute the gradient of the loss function with respect to the model parameters using the mini batch.
4. Update the model parameters with a small step in the direction of the negative gradient.
5. Repeat steps 2–4 until the model converges or a stopping criterion is met.

The key advantage of SGD is that it can converge faster than batch gradient descent, especially for large datasets. However, the convergence may not be as smooth, and the optimal solution may not be reached due to the stochastic nature of the algorithm.

(1) E(w,b)=\frac{1}{n}\mathop{\sum }\limits_{i=1}^{n}L({y}_{i}f({x}_{i}))+\alpha R(w).]

**Figure 1:**
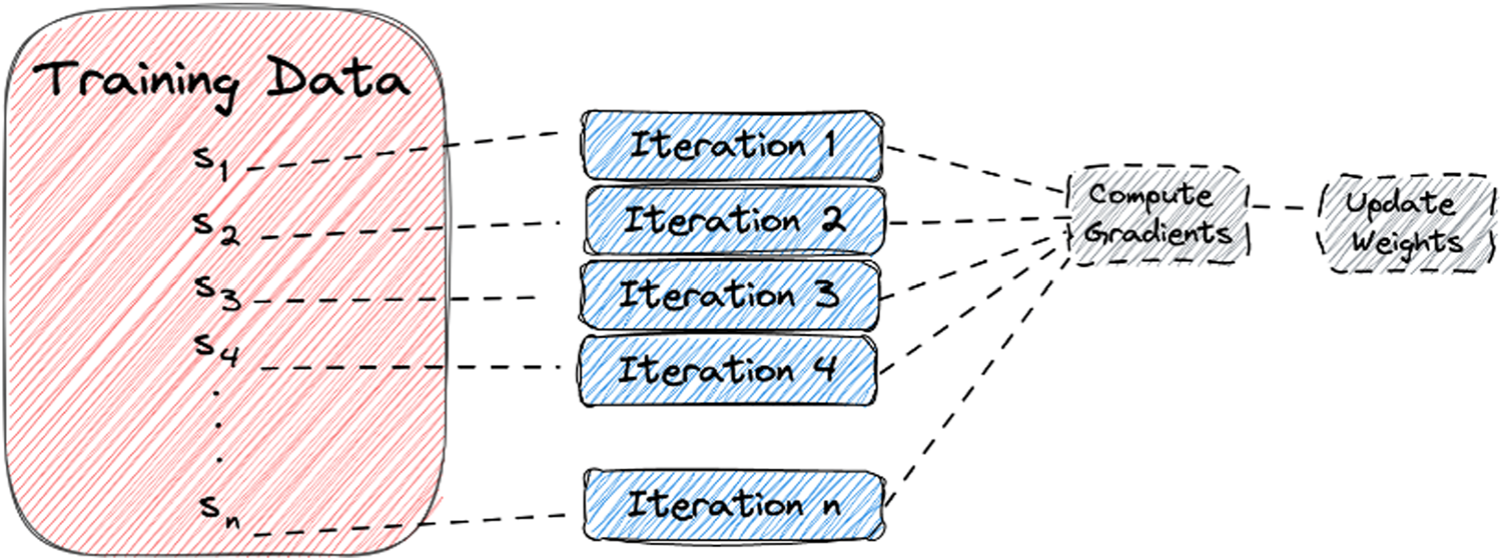
Schematic of SGD approach [3].

Figure 1 depicts the procedure of the stochastic gradient approach operation. With estimate of the applied knowledge in application of stochastic medium built with gradient descent optimisation because substitutes to the original division of gradient analysis through estimation strategy. For one-class optimisation problems,

(2) \mathop{{\rm{\min }}}\limits_{w,\rho ,\xi }\frac{1}{2}{||\omega ||}^{2}-\rho \xi +\frac{1}{{v}^{n}}\mathop{\sum }\limits_{i=1}^{n}{\xi }_{i.}]

For w\forall \in 0,]

(3) {y}_{i}=\rho +\frac{w}{b}\xi -\frac{1}{{v}_{i}}\mathop{\sum }\limits_{n=0}^{i}{\rm{\max }}(0,\rho -(w,{x}_{i})),]

(4) \mathop{{\rm{\min }}}\limits_{w,\rho ,\xi }\frac{v}{2}{||\omega ||}^{2}+{bv}\xi -\frac{1}{n}\mathop{\sum }\limits_{i=1}^{n}{\rm{\min }}((w,{x}_{i})+b),\hspace{1em}]

(5) R(w)=\frac{1}{2}\mathop{\sum }\limits_{j=1}^{m}{w}_{j}^{2}={{\rm{|}}|w|{\rm{|}}}_{2}^{2},]

(6) R(w)=\mathop{\sum }\limits_{j=1}^{m}{{\rm{|}}w{\rm{|}}}_{j},]

(7) R(w)=\frac{\rho }{2}\mathop{\sum }\limits_{j=1}^{n}{w}_{j}^{2}+(1-\rho \xi )\mathop{\sum }\limits_{j=1}^{n}{|w|}_{j}.\hspace{1em}]

It reduces the computational burden, permitting booster iterative exchange over the approach made among the decreased convergence rate, particularly with the problems concerned with high-dimensional optimisation difficulties, and provides a good overview of convergence rates. Given a set of training examples ( {x}_{1},{y}_{1}] ), … ( {x}_{n},{y}_{n}] ), where {x}_{i}\in {R}^{m}] and {x}_{i}\in R] ( {y}_{i}\in -\mathrm{1,1}] for classification approach) that aims for the scoring basis into the function generated by the linear systems scored for a minimal range of f(x)={w}^{T}x+b] integrated under specific parameters of the model based on intercept built at b\in R] . To create estimate values with a binary classification approach that simplifies within sign functionality allotted to f(x)] . For model identification in the model parameters, training regularised into the erroneous regularised built with the formula equated as per the analysis is minimised.

For one-dimensional (1D) variables, SGD works by iteratively adjusting the value of the variable based on the gradient of the loss function with respect to the variable. The working is as follows:

1. Initialise the variable with a random value.
2. Compute the gradient of the loss function with respect to the variable at the current value of the variable.
3. Update the variable by subtracting a small multiple of the gradient from the current value. The multiple is called the learning rate, and it determines how quickly the algorithm converges to the optimal value.
4. Repeat steps 2 and 3 until the algorithm converges to the optimal value.

In SGD, step 2 is performed using a randomly selected subset of the data, rather than the entire dataset. This makes the algorithm more efficient and allows it to scale to large datasets.

(8) \omega \to \omega +\eta \left[\alpha \frac{\delta R\left(w)}{\delta w}-\frac{\delta L({w}^{T}{x}_{i}\left+{b}_{i}{y}_{i})}{\delta w}\right],]

(9) {\eta }_{(i)=\frac{{et}\alpha }{t},}]

(10) {\eta }_{(i)=\frac{1}{\alpha (t+{t}_{0})}.}]

**Figure 2:**
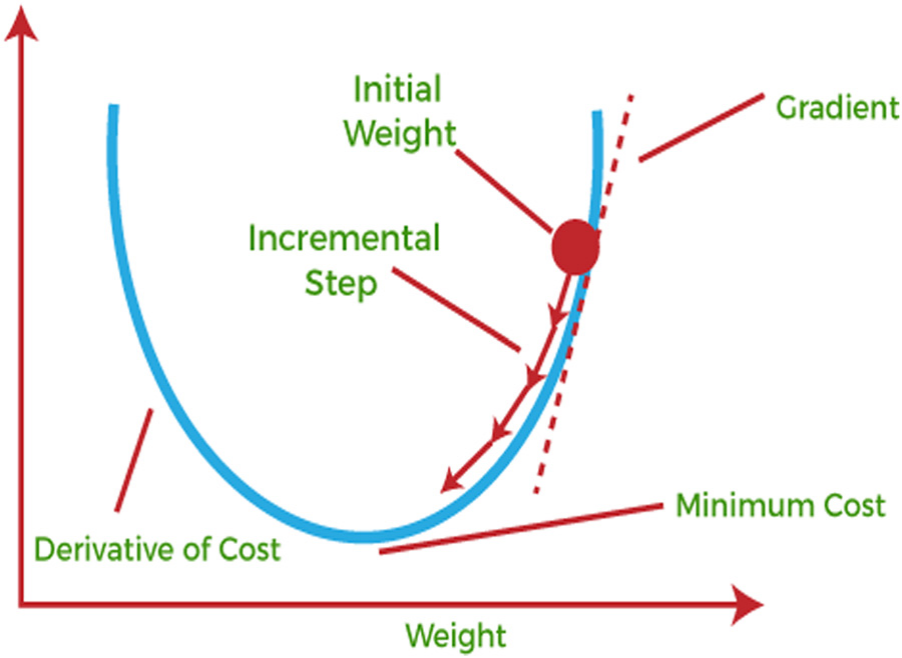
Gradient descent works in the case of one-dimensional variables [7].

**Figure 3:**
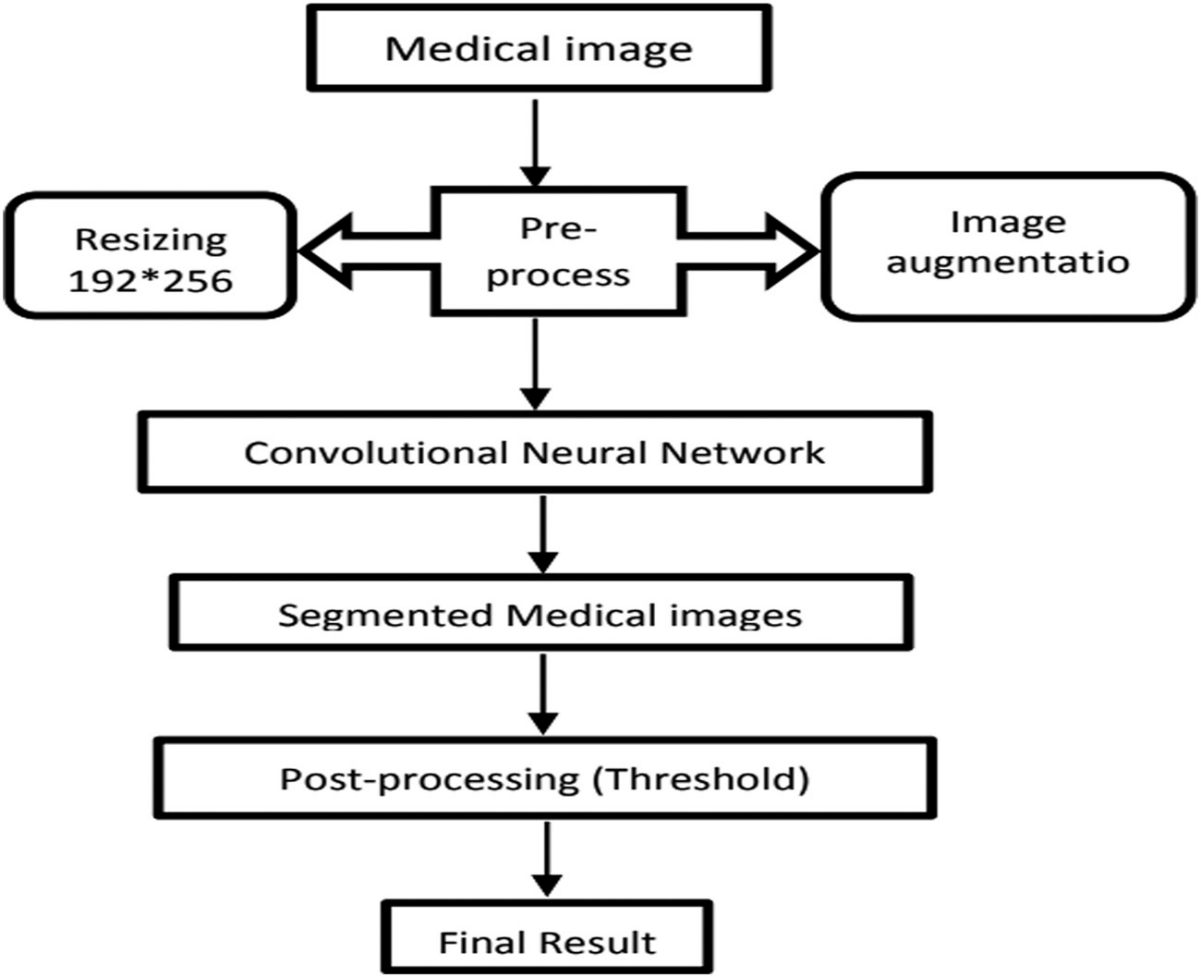
Sequence of the implemented algorithm.

Figure 2 depicts the operation of gradient descent work through the 1D variable approach. This method includes two pre-processing steps on medical images that feed into the network. These steps include image resizing and image augmentation. Following that, pre-processed images are sent to the network for segmentation. For model identification in the model parameters, training regularised into the erroneous regularised built with the formula equated as per the analysis is minimised. The results are blurred segmented images. We solved this issue by employing a post-processing step that uses a threshold technique to sharpen the segmented images. Our algorithm’s sequence has been demonstrated in Figure 3.

Figure 3 depicts the sequential process of implemented technique with the analysis built with the process. For model identification in the model parameters, minimisation of training regularised into the erroneous regularised created with the formula equated as per the analysis. Following that, pre-processed images are sent to the network for segmentation.

## 4 Network architecture

The proposed deep neural network has an encoder–decoder structure with 64 layers (excluding the final activation layer). Each encoder sequence has attained numerous convolutional layers under batch normalised integrated to ReLU nonlinearity, observed through a sampling technique designed with the non-overlapping case for the max pooling and sub-sampling strategy. Before initial sampling begins, two dense layers are present in the network’s centre. The max pooling indices distinguish the proposed network in the performance analysis upon generating the resolution into mapping the case of featured research. It results in the retaining way to feature details for removing the non-useful features. This neural network produces smooth images without the use of any post-processing techniques. This method has the potential to outperform two FCN and U-net architectures. Despite using up-convolutional layers and a few shortcut connections, FCN generates coarse segmentation maps. As a result, more shortcut connections are added. This proposed method uses less memory than FCN. In contrast to FCN, indices from max pooling are copied rather than encoder features. Also, the outputs produced by this method are smooth, and the borders between the ROI and the surrounding areas cannot be detected precisely by the physicians; as a result, we used a threshold technique as a post-processing technique to sharpen the results, as explained in the Sections 4.1 and 4.2.

**Figure 4:**
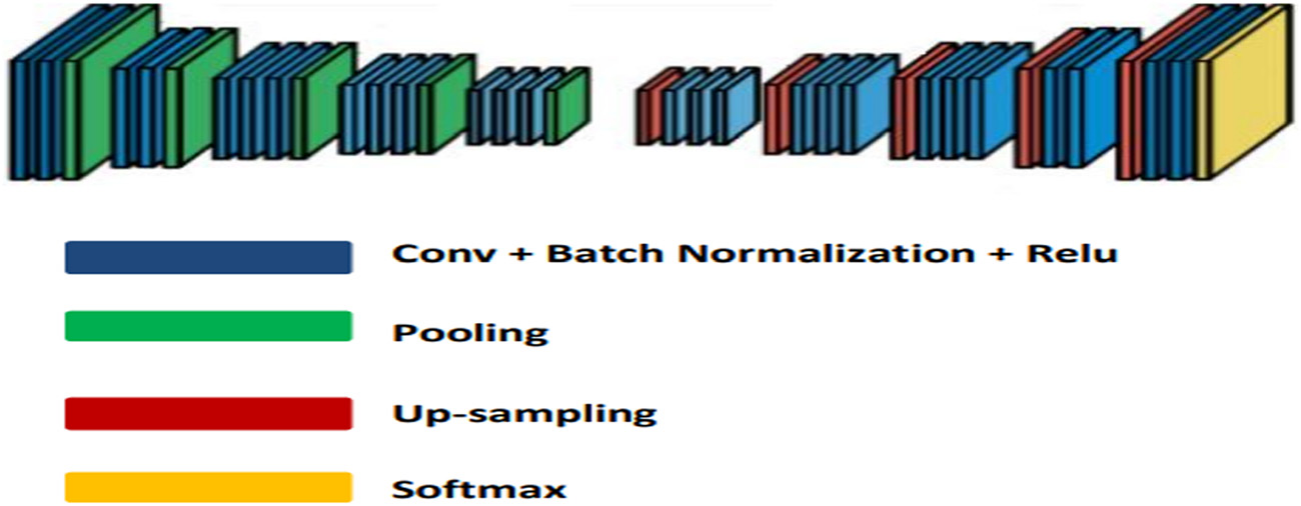
Architecture of the proposed neural network.

Figure 4 depicts the proposed neural network architecture. The architecture comprises a consecutive approach with nonlinear processing layers under encoding and decoding based on a classifier of pixel-wise strategy. The individual encoder can be composed based on layers convoluted with the method of batch normalisation along with ReLu with the nonlinearity approach being overlapped with the sampling process that underwent non-overlapping oriented over max pooling and sub-sampling scheme. In accordance with sparse integration, the decoding parameter can be developed by subjecting the encoding process to the unsampled case of decoding and using indices sequenced under maximum pooling with performance for highlighting the mapping process as a key case in developing the count reduction in favour of the details frequency in the images. The frequency split into the images for which the most significant analysis during the decoding process linked with the details took place was through approaching a qualitative basis at the end process.

### 4.1 Loss function

A loss function is a mathematical function that helps to evaluate how well a machine learning model is performing. It compares the predicted output of the model with the actual output and calculates the difference between the two. The goal is to minimise this difference in order to improve the accuracy of the machine learning model. There are many different types of loss functions, each with their own strengths and weaknesses, depending on the type of problem you are trying to solve. Both binary cross-entropy and Jaccard distance are loss functions in this case. The cross-entropy is a function that calculates how far the prediction is from the actual value for each class and then averages the class of the error by class to calculate the final loss. In this problem, each pixel has only two categories: black or white (0 or 1), depending on the mask. As a result, rather than the categorical cross-entropy originally proposed, binary integrated to cross-entropy as per loss function is utilised. The expressed formulation of binary-oriented cross-entropy was signified based on

(11) l(y,\hat{y})=-\frac{1}{N}\mathop{\sum }\limits_{i=0}^{N}(y* {\rm{\log }}({\hat{y}}_{i})+(1-y)* (1-({\hat{y}}_{i}))).\ ]

### 4.2 Network training

**Figure 5:**
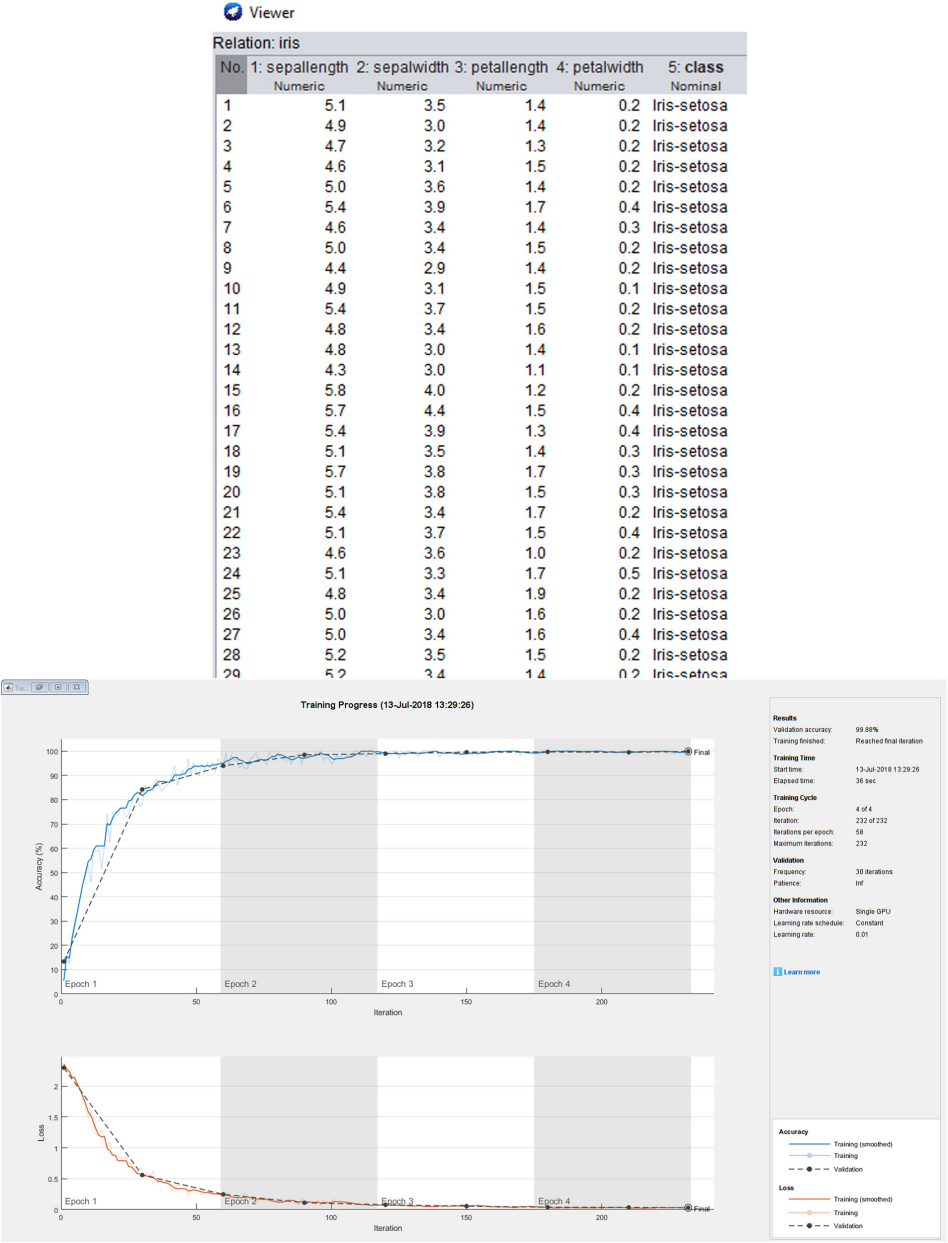
Training, testing, and validation of CNN.

We used 75% of the 200 images in the PH^2^ dataset for the training process [23]. Furthermore, the actual image count in PH^2^ dataset together with chest-Xray images is more significant than 150 because the number of images increases to 450 after image augmentation as pre-processing and then enters the network. We separated these images into training and validation datasets to improve network performance. 20% of these 450 images are associated with the validation dataset, while the remaining are related to the training dataset. Figure 5 depicts the network configurations of training, testing, and validation of CNN. Based on the architecture developed within the network, parameters sum up under the networking process that undergoes training steps of 33,486,874 out of 33,384,879, under the summation of non-trainable parameters of 15,982. The implementations are written in Keras, and the environment is Google.

## 5 Image augmentation

**Figure 6:**
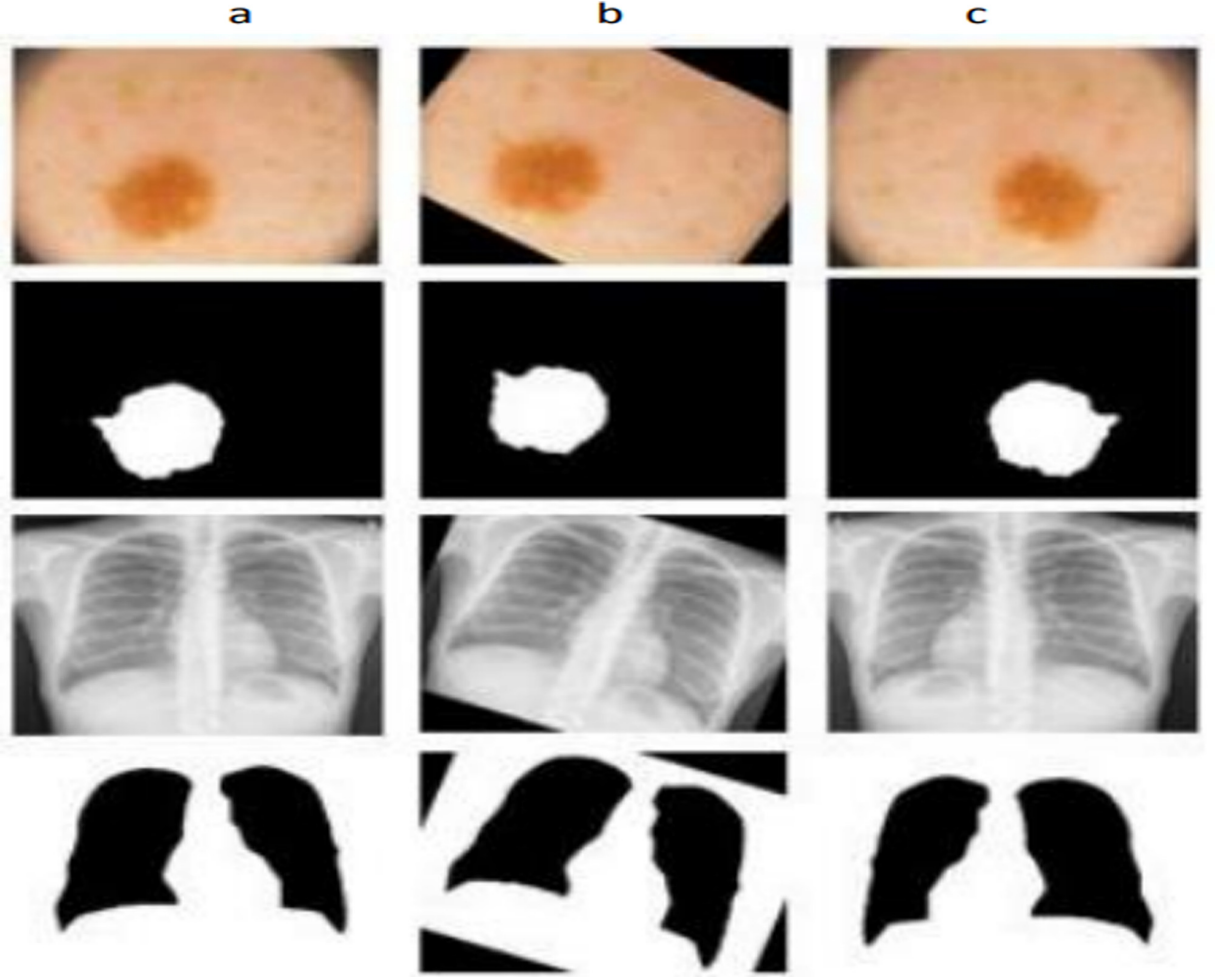
Image augmentation technique. (a) original medical images with their corresponding masks. (b) Rotated images with their corresponding masks. (c) Horizontally flipped images with their corresponding masks.

Image augmentation on training images was used to improve the model’s robustness and reduce the likelihood of overfitting. It will also enhance the image count in the dataset available within the source. Image rotation and horizontal flipping are two simple techniques used. The images are rotated around the (−40, +40) degrees in image rotation, and the images are reversed around the horizontal axis in a horizontal flip. To maintain exact orientation with the image featuring a mask of true nature throughout, the strategy transformed with a critical image positioned into the correspondent precise. As stated in Section 4, performance of the images under the count of 200–500 will improvise once the processing of training methods gets implemented. On average, 30% of the datasets accessible for a certain condition have a collection of training photos for validating groups connected with those datasets. Figure 6 shows the technique in which the image is segmented with the open data source.

### 5.1 Batch normalisation

Batch normalisation is a technique for speeding up the neural network learning process by normalizing the values in the hidden layers, like the principle behind normalizing features in data or activation values. The batch normalisation layer is presented after each convolution layer in the proposed network for 25 batch normalisation layers in the entire network architecture.

## 6 Experimental results and design

The medical datasets used include the PH^2^ dermoscopic dataset, which contains 200 dermoscopic images and their label masks, and the chest-Xray images, which contain 200 RGB images and their label masks. The dataset has been developed based on the experimental investigation processed to facilitate image segmentation and classification. Dermoscopic images have a resolution of 572 × 765, while chest-Xray images have a resolution of 512 × 512. Besides, the PH^2^ dataset was obtained from the Dermatology Service of Hospital Pedro Hispano in Matosinhos, Portugal, and the chest-Xray dataset was made available in Kaggle. With two pre-processing before feeding the medical images to the network, one of which is resizing the images to speed up the neural network, in this work, we resize and reduce the size of all medical images to 192 × 256. It significantly diminishes, as well as training time in addition to complexity, without affecting the results significantly. They also utilised RGB 8-bit images for training and evaluating our model and labelling masks to test it. The evaluation set is 20% the size of the training set, and the test set is 25% of the scope for the label mask set (50 images).

### 6.1 Characteristic procedures for quality evaluation for image integration methods

Image Fusion techniques’ performance is measured using a variety of metrics. The combining image procedure preserves general information by inputting images while avoiding introducing unwanted facts. The proposed neural network’s outputs are binarised into lesion masks. The proposed algorithm’s performance was evaluated by comparing computer-generated masks as output to ground truths from the label masks dataset. Performance evaluation is performed for the model developed based on the following metrics:

Assume TP, FP, TN, and FN indicate true positives, false positives, true negatives, and false negatives count.

- Dice coefficient: Like the precision rate, Dice score (*F*1) means that it quantifies positive analysis that applies consequence within the false positive within the model development. It is more like precision than accuracy. We have shown the dice coefficient by DI and have calculated it by the following formula:
{\rm{DI}}=\frac{2* {\rm{TP}}}{({\rm{TP}}+{\rm{FP}})\left+({\rm{TP}}\left+{\rm{FN}})}.]
- Recall: Gives the measure that targets positive states under actual natures that have yielded through the output model. Within the cases of economic analysis with a false negative claim superior to recall, the metric chooses the best model among the possible ones. Recall is also known as sensitivity (SE). We can calculate Recall using the following formula:
{\rm{SE}}=\frac{{\rm{TP}}}{{\rm{TP}}+{\rm{FN}}}.]
- Accuracy: Accuracy approximates how close our predicted output is to the absolute ground truth. It is denoted by AC and is calculated using the following formula:

{\rm{AC}}=\frac{{\rm{TP}}+{\rm{TN}}}{{\rm{TP}}+{\rm{FN}}+{\rm{TN}}+{\rm{FP}}}.]

## 7 Comparison with two famous architectures

We demonstrated our findings using chest-Xray medical pictures and compared them to other methods. Furthermore, we compared our results with two well-known architectures, U-net and FCN networks, and strategies implemented by Yuan et al. and Gutman et al. [24,25]. In addition, we used the chest-Xray dataset to train the algorithm on different medical images. Table 1 displays our results from the PH^2^ dataset.

**Table 1:** Comparison results with other methods, based on different optimisation algorithms, using the PH^2^ dataset

Method	Sensitivity	Accuracy	Dice coefficient
			
FCN	85.6	97.3	62.0
U-net	93.2	91.1	65.6
The proposed method	97.2	98.1	77.3

Table 1 lists the comparative results with the other methods, based on different optimisation algorithms, using the PH^2^ dataset, which has robust results in medical image segmentation and has been used in many works.

We compared our method with the U-net and FCN methods, which have robust results in medical image segmentation and have been used in many works. The developed method produces better results with higher evaluation metrics than the other methods we discussed. Table 2 lists the comparative results with other methods, based on different optimisation algorithms, using the chest-Xray dataset, which has robust results in medical image segmentation and has been used in many architectures built.

**Table 2:** Comparison results with other methods, based on different optimisation algorithms, using the chest-X-ray dataset

Method	Sensitivity	Accuracy	Dice coefficient
FCN	96.7	96.5	96.8
U-net	95.6	94.2	95.9
The proposed method	99.3	98.87	98.63

**Figure 7:**
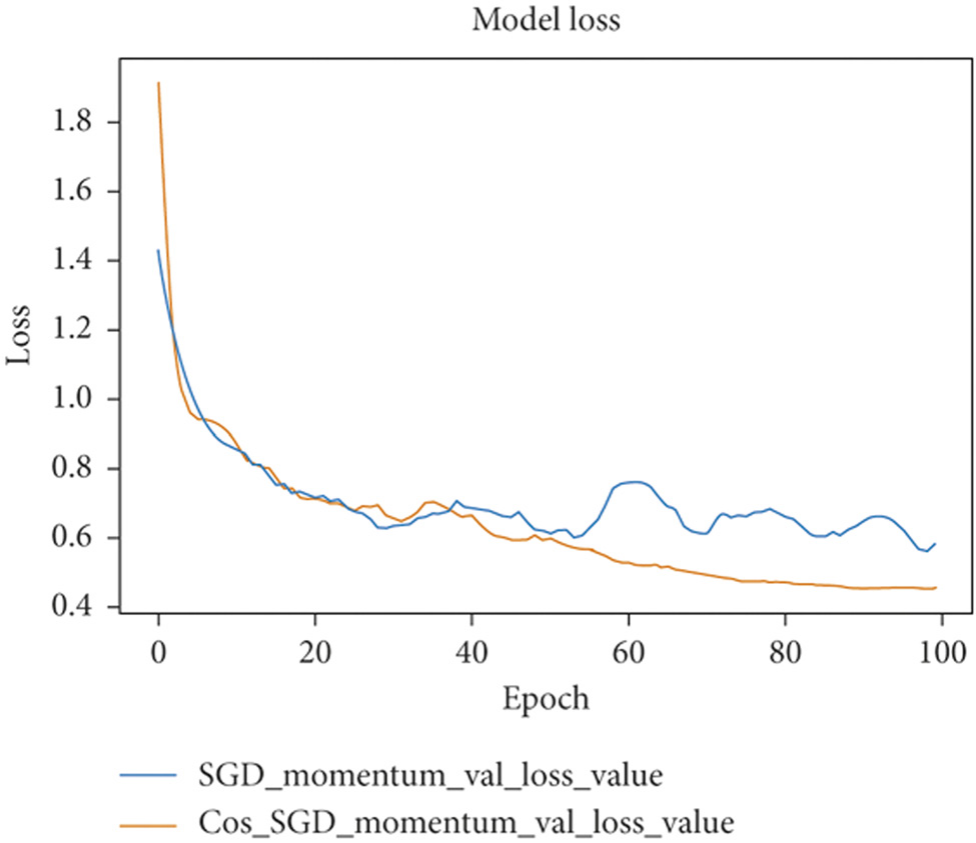
Estimation of model loss.

Figure 7 depicts the estimation of model loss. In addition, we implemented the segmentation of original images in the results. In our algorithm, we used a post-processing step that uses a threshold technique to increase the resolution of segmented images and sharpen the borders between ROI and other regions. Using this technique, we can compare the segmented outputs to their corresponding binary masks and observe the performance of our algorithm.

**Figure 8:**
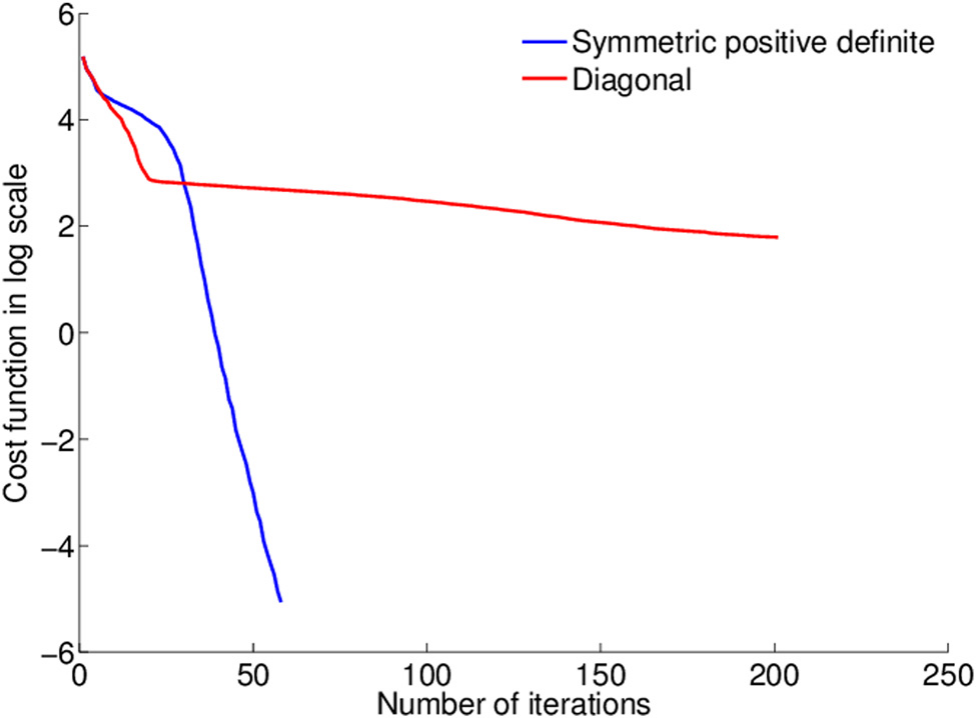
Convergence effect of SGD strategy.

Figure 8 illustrates the convergence effect of SGD strategy. The ROI boundaries are clear, and specialists can detect them completely. For dermoscopic images, there are numerous complex ROIs with varying illumination that our method was able to overcome and segment the regions in these images.

## 8 Results and discussion

**Figure 9:**
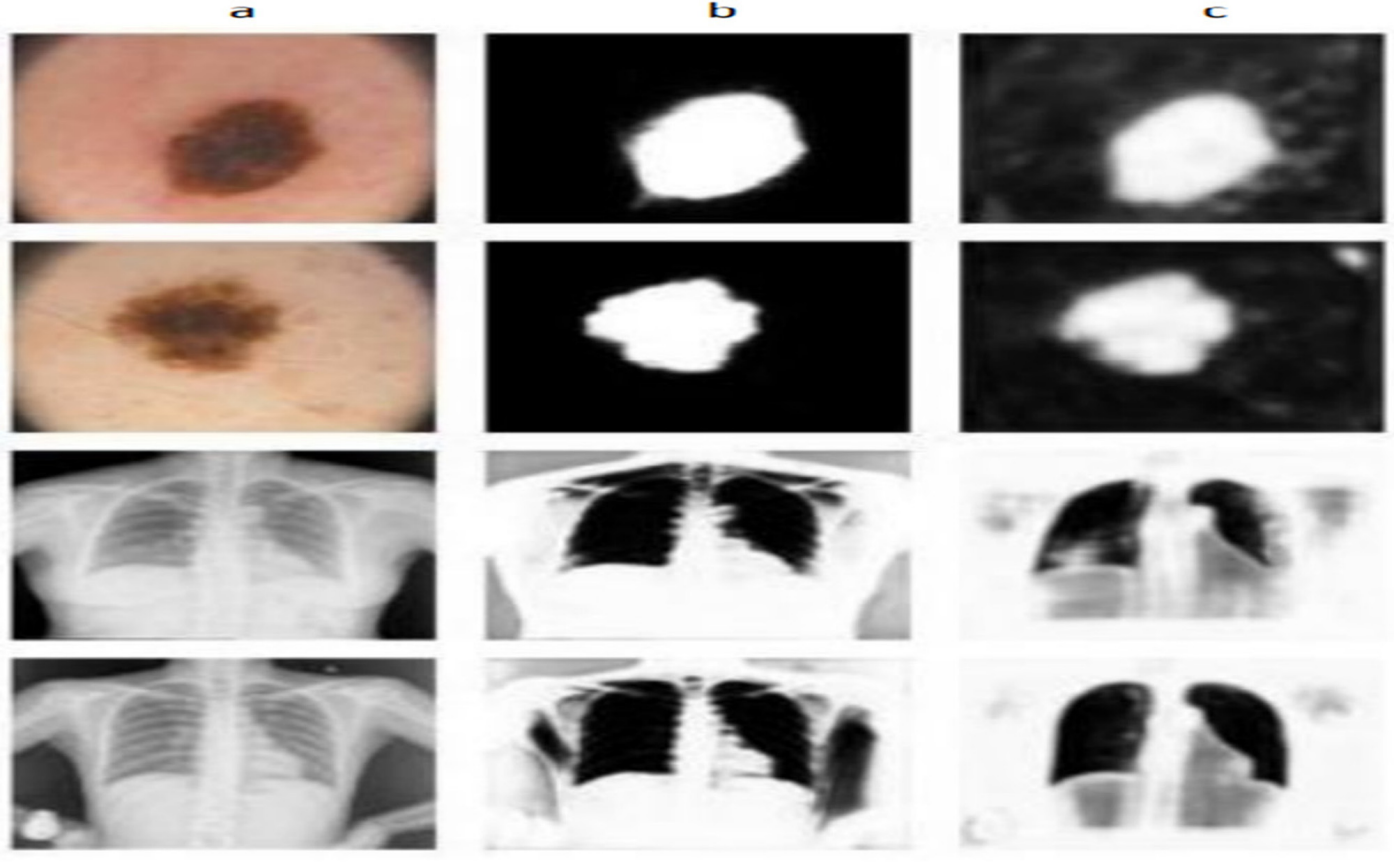
Results of U-net and FCN. (a) Original medical images. (b) U-net segmentation results. (c) FCN segmentation results.

We implemented our algorithm in the Google collab space, and our simulation results are shown in Figures 5 and 6. We implemented our results in matlab2014 through a segmentation application with the images of medical practice. Our model performs well with blurry borders and segmented skin lesions. On the contrary, the boundaries of the ROI are evident in the results, and doctors can completely recognise the ROI and cure the patients. Furthermore, we applied two FCN and U-net methods to our dataset, but as shown in Figure 4, the ROI borders are ambiguous and unclear. Our method is a robust and precise medical image segmentation algorithm that can be applied to other medical images such as brain cancer datasets, bladder, Covid19-chest-Xray medical images, etc. This method shows each medical image’s segmentation and results. First, in Figure 5, we offer the results for dermoscopic medical images. Comparatively, the proposed technique is more robust than the remaining approaches based on the outcomes. The ROI boundaries are clear, and specialists can detect them completely. For dermoscopic images, there are numerous complex Regions of Interest with varying illumination that our method was able to overcome and segment the regions in these images. There are also illumination variations for chest-Xray images and our algorithm could overcome these problems too. Figure 9 shows the results of U-net and FCN based on the image segmented with the developed process.

**Figure 10:**
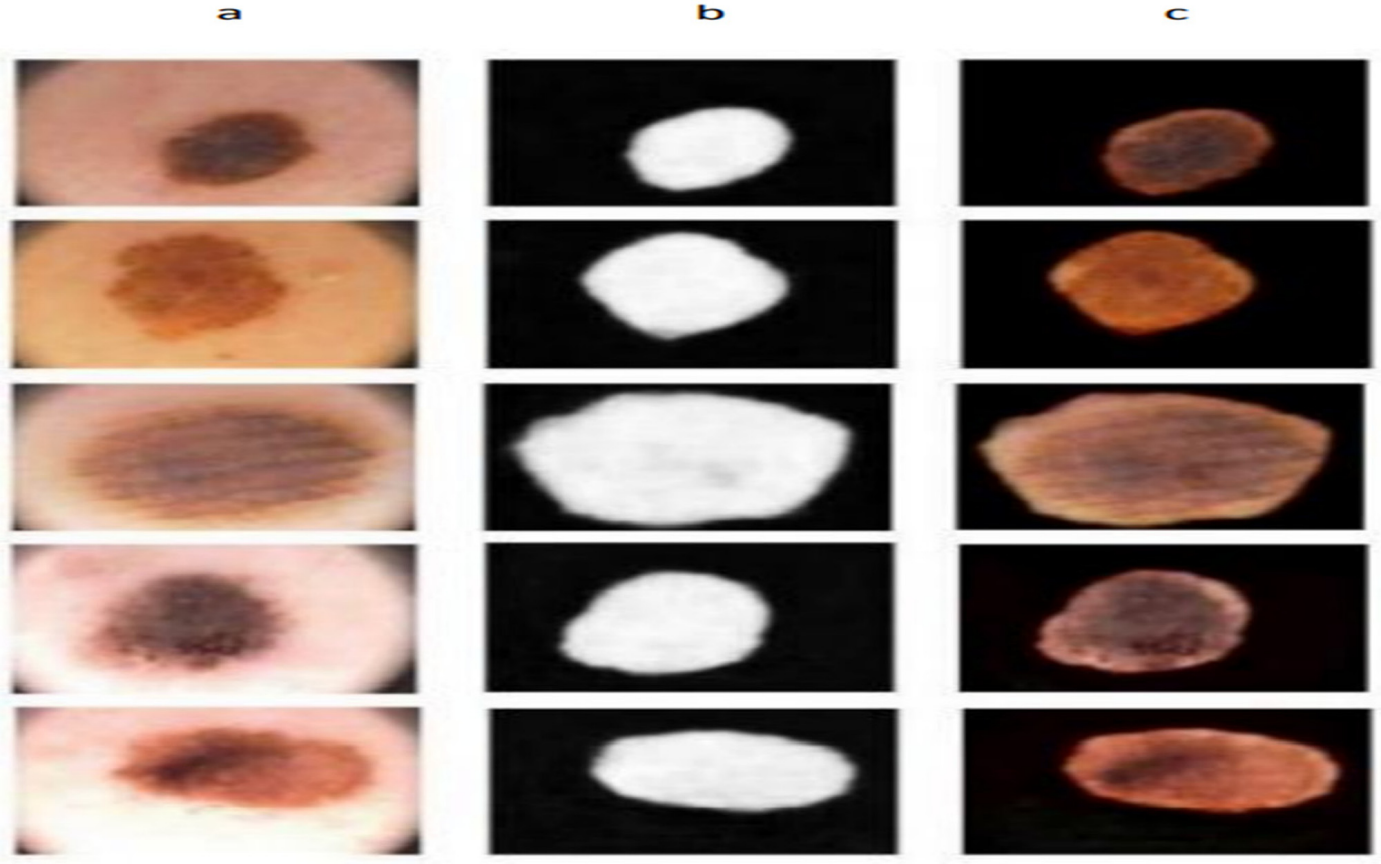
Final simulation results. (a) Original dermoscopic images. (b) Segmentation outputs. (c) Final results.

**Figure 11:**
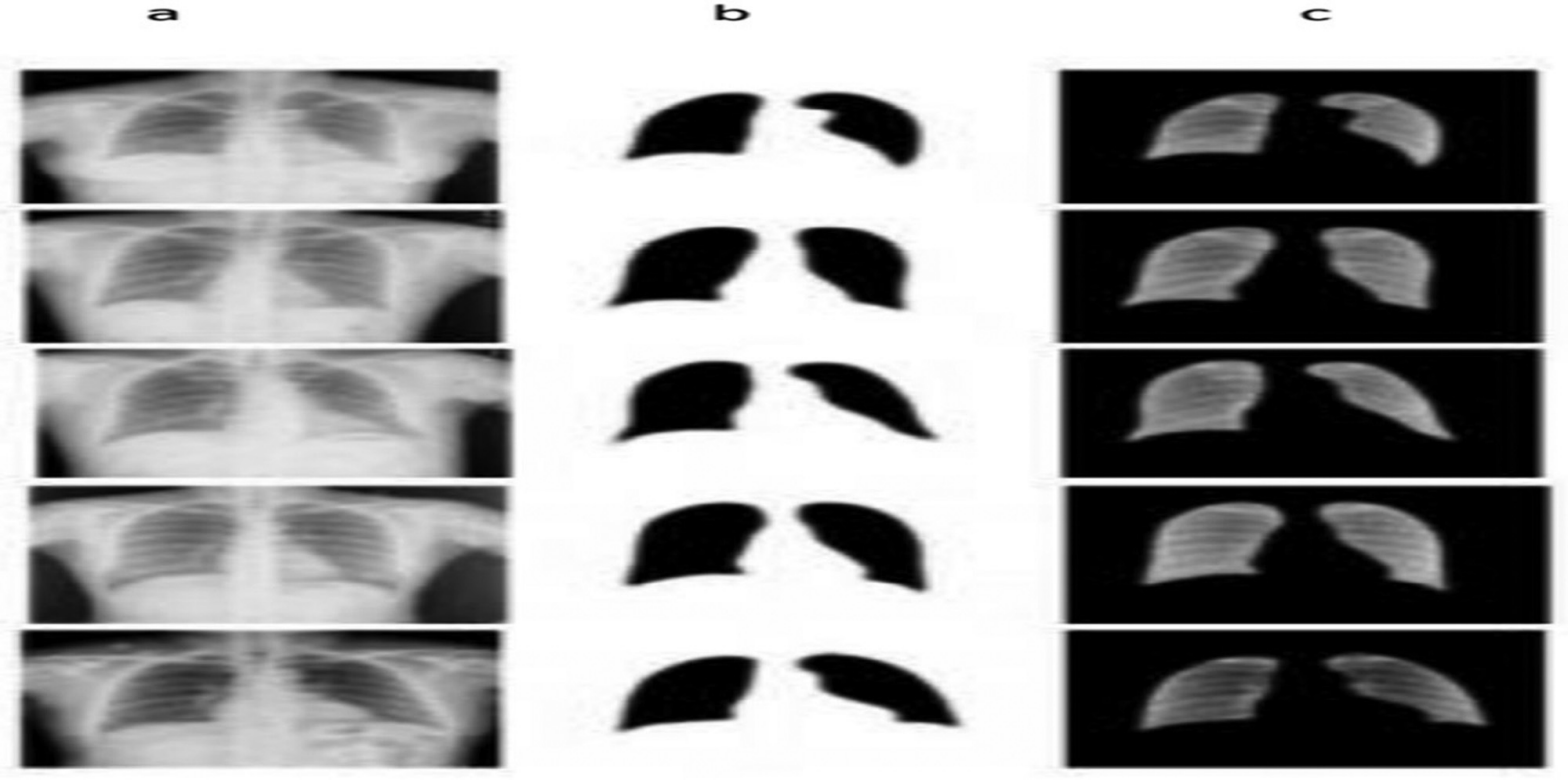
Final simulation results. (a) Original chest-Xray images. (b) Segmentation outputs. (c) Final results.

**Figure 12:**
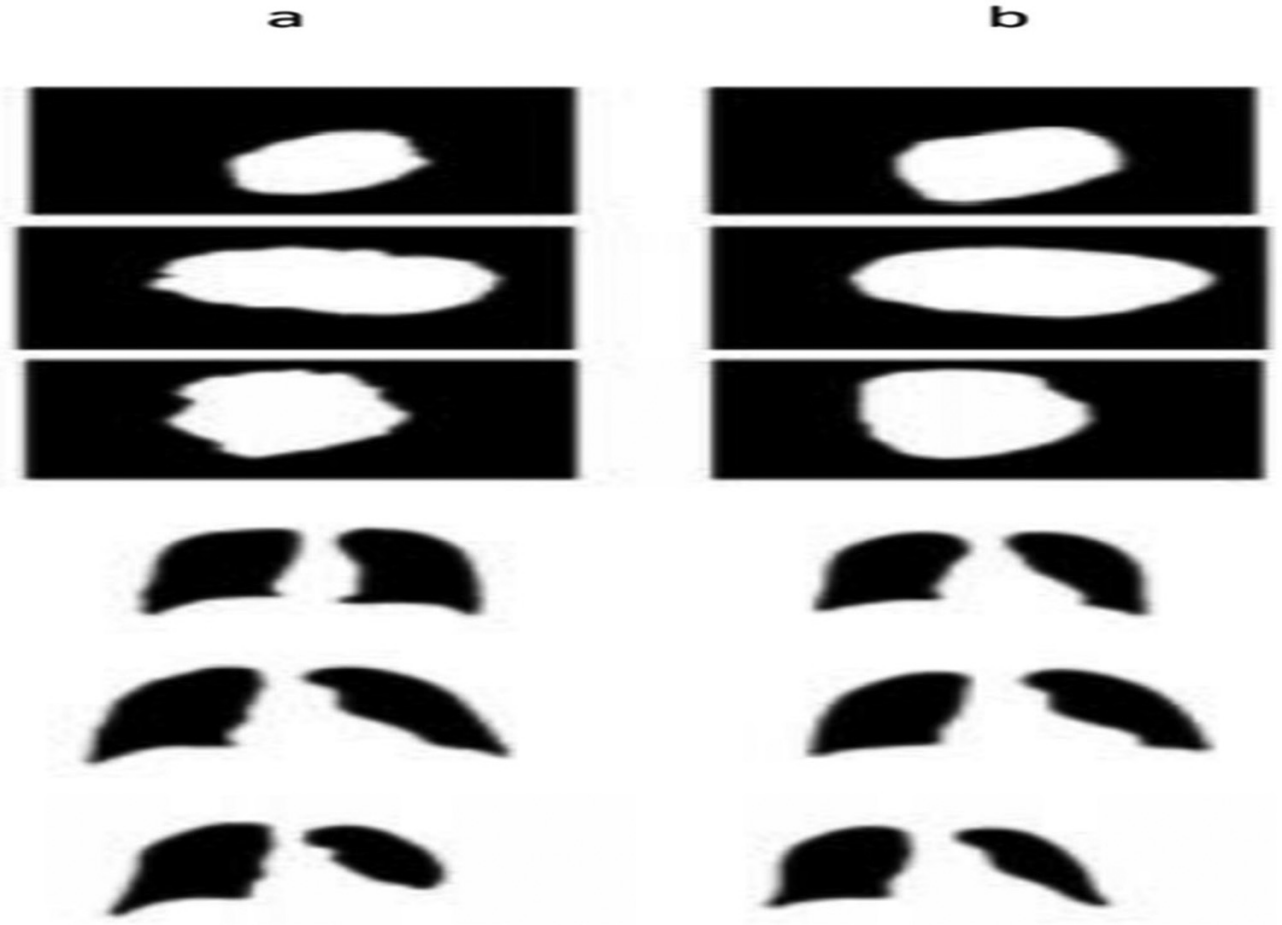
Post-processed outputs. (a) Binary masks as ground truths. (b) Corresponding segmented outputs of each binary mask after post-processing.

Figure 10 depicts the final simulation outcomes with the evaluation verified through the process identified under segmentation for dermoscopic images. Figure 11 illustrates the final simulation outcomes with the assessment verified through the process identified under segmentation for chest-Xray images. Figure 12 shows the post-processed results with the truth mask built with the ground analysis of binary classification. Further, segmented and separated the texture of the lung from other parts of the chest-Xray medical images, and the Chest bone structures are visible in these images. This work can detect all problems, including lung tumours, lung or bone structure lesions, lung infections, and diseases caused by them, such as Covid19. According to the results, the proposed algorithm produces better and more accurate results than U-net and FCN. The borders between lungs and other textures in the segmented images are identical to those in the original images. In addition, we implemented the segmentation of authentic images in the results. In our algorithm, we used a post-processing step that uses a threshold technique to increase the resolution of segmented images and sharpen the borders between ROI and other regions. Using this technique, we can compare the segmented outputs to their corresponding binary masks and observe the performance of our algorithm.

## 9 Conclusion

This study proposed a novel deep-CNN-integrated methodology for applying medical image segmentation upon chest-Xray and dermoscopic clinical images. The research study understands the various strategies involved in the training format concerning the issues faced in deep neural networks with data sources of limited scenarios. Our loss function was created using both Jaccard distance and binary cross-entropy. Before feeding the training dataset to the network for training, the augmented set of image processing fields is underutilised. Besides the performance of specific actions on images that include rotation along with flipping moment horizontally, the technique is validated under testing aspects within the evaluated model of the network to apply statistical predictions. The projections facilitating the model for images under testing undergo a post-processing approach to meet the threshold case of diminishing the boundaries with sharpened images for better detecting the issue. All the test cases were operated at no constraint and within the data fed with Skin Lesion Analysis Towards Melanoma Detection in PH^2^ but rather chest-Xray medical images. The technique outperformed conventional or advanced approaches, including FCN and U-net. Results validated are compared, and it is found that the novel method is robust and reliable for detecting various conditioned images under artefacts within data acquisition through minimal processing practice. Thus, through the outcomes, it is straight to say that the technique is quite applicable for any segmentation tasks in the medical practice. The performance measures obtained in our experiments are Sensitivity = 0.9913, Accuracy = 0.9883, and Dice coefficient = 0.982. Also, future research will further develop our method for dice coefficient improvement and apply it to other well-known datasets, such as ISIC2017 and ISBI2016, as well as other medical datasets for numerous body parts.

Future scope of the research study includes adaptive learning rates, mini-batch selection strategies, regularisation techniques, parallel and distributed computing, loss function design, transfer learning, uncertainty estimation, and hardware acceleration. Advancements in these areas can lead to improved segmentation accuracy, faster convergence, and more efficient deployment of CNN models in medical imaging applications.
